# Experimental analysis of the dynamical response of energy harvesting devices based on bistable laminated plates

**DOI:** 10.1007/s11012-015-0140-1

**Published:** 2015-03-13

**Authors:** A. Syta, C. R. Bowen, H. A. Kim, A. Rysak, G. Litak

**Affiliations:** 1 Faculty of Mechanical Engineering, Lublin University of Technology, Nadbystrzycka 36, 20-807 Lublin, Poland; 2 Department of Mechanical Engineering, University of Bath, Bath, BA2 7AY UK

## Abstract

The use of bistable laminates is a potential approach to realize broadband piezoelectric based energy harvesting systems. In this paper the dynamic response of a piezoelectric material attached to a bistable laminate plate is examined based on the experimental generated voltage time series. The system was subjected to harmonic excitations and exhibited single-well and snap-through vibrations of both periodic and chaotic character. To identify the dynamics of the system response we examined the frequency spectrum, bifurcation diagrams, phase portraits, and the 0–1 test.

## Introduction

Recently, various energy harvesting devices have been developed in an attempt to convert ambient vibrations to electrical energy [1, 2]. This interest has stemmed from the need to develop autonomous low-powered electronic systems such as wireless sensor networks and safety monitoring systems. For vibration harvesting the use of piezoelectric materials is a potential route for generating the necessary power levels, typically in the $\upmu \hbox {W}$ to mW range. The advantages of these materials are their higher strain energy densities compared to electrostatic and electromagnetic systems and their ease of integration with mechanically vibrating structures [3].

In many cases, such as those on railway carriages [4] or other forms of transport [5, 6], the ambient vibrations can exhibit multiple time-dependent frequencies, may change with time and can include components at relatively low frequencies. It has been reported that introducing nonlinear effects can lead to an improvement of the frequency bandwidth of the vibration energy harvester [7].

As a result, a variety of approaches for incorporating non-linearity in the stiffness of energy harvesters have been considered, most notably by designing bistable harvesters with two distinct energy wells [8–13] using repulsive or attractive magnetic interactions between a cantilever and an external magnet, axial loading of canilevers and the use of post-buckled beams.

An alternative method of developing bistability was reported by Arrieta et al. [14–18] where a piezoelectric element was attached to an asymmetric bistable laminate plate made from a carbon fibre reinforced polymer (CFRP) laminate with a [0/90]$_T$ layup. Due to the difference in the coefficient of thermal expansion between the carbon fibre and epoxy matrix the thermal residual stress developed on cooling of the laminate from an elevated cure temperature leads to it exhibiting two distinct stable states. When subjected to large amplitude oscillations the laminate undergoes snap-through between the two stable states. For energy harvesting [19] when a piezoelectric material is attached to the bistable laminate surface it can generate power by repeated straining as it experiences deformation as a result of mechanical vibrations. Experimentally, such harvesting devices have been shown to exhibit high levels of power extraction over a wide range of frequencies when harmonically excited from a central mounting [20], with the scope for improved power generation through changes in the geometry. The potential advantages of using the intrinsic thermal stress in the laminate to induce bistability, compared to using magnetic configurations [19, 21] is that (1) the laminate can be designed to occupy a smaller space and there are no stray magnetic fields, (2) the laminate can be readily combined with piezoelectric materials and (3) there is potential to tailor the laminate lay-up, laminate elastic properties and geometry to provide additional control over the harvester response to the vibrations that are being harvested.

In the present work we employ an electro-mechanical system to generate mechanical vibrations leading to snap-through of the laminate between its two stable states. Such a system has the potential of have a broadband frequency response in terms of its voltage output. At this stage it is of interest to note that a monostable system is characterized by a single potential well while a bistable by a double potential well. In contrast to the simplest mono-stable linear system, which shows narrow frequency resonance, bistable structures are inherently nonlinear and are characterized by an inclined (nonsymmetric) resonance curve covering the wider region of frequencies. Another effect caused by strongly nonlinear bistable system can be the appearance of multiple solutions. In such a case, the solutions can be grouped into the hopping cases with large amplitudes and those sitting in the single potential well with small amplitude of oscillations. The advantage of a bistable resonator is visible for lower frequencies. In our system of a bistable plate, hopping between potential wells is realized by a snap-through phenomenon. Due to the linear coupling between displacement and voltage in a piezoelectric patch, a larger vibration of amplitude response for given excitation frequency implies larger power output.

The motivation of this work is to develop methods to identify the bistable mechanical resonator response to vibrations; these include single well oscillations, continuous snap-through between stable states and the existence of chaotic or periodic snap-through behaviour [20, 22]. An understanding of the nature of the complex dynamic response of such a system could be used to optimise the ambient vibration energy harvesting.

## Experimental setup

**Fig. 1:**
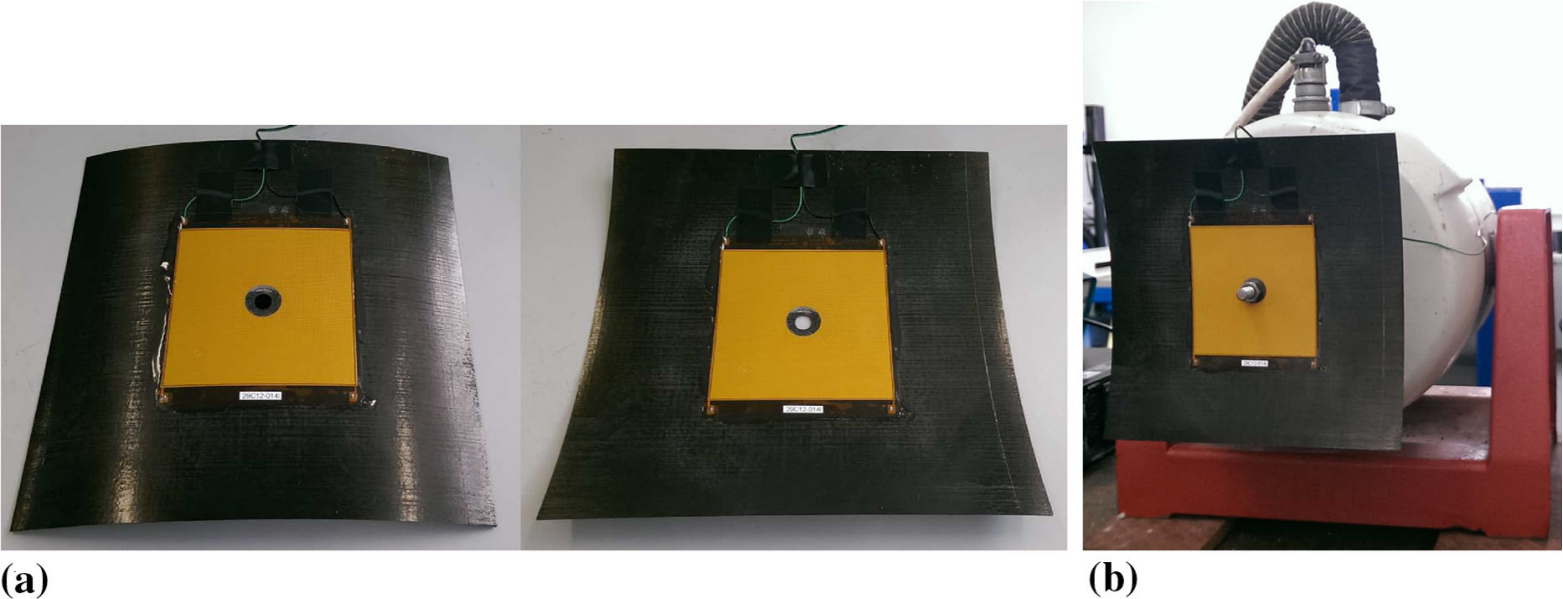
**a** The laminate used for these tests measures 190 mm × 190 mm × 0.5 mm, has a [0/90]$_T$ layup and has a single piezoelectric layer Micro-Fibre-Composite (MFC) attached to the top surface of dimensions 85 mm × 85 mm × 0.3 mm, and **b** experimental setup showing mechanical shaker attachment

A square [0/90]$_T$ carbon fibre reinforced laminate was considered as the basis for developing a broadband energy harvesting device. The laminate measured 190 mm by 190 mm and was made from M21/T800 CFRP prepreg material. A single piezoelectric Macro Fiber Composite (MFC) layer (M8585-P2, 85 mm × 85 mm) was bonded to the laminate surface. Figure 1a shows the two stable state of the CFRP-MFC combination which is mounted to an electrodynamic shaker (LDS V455) at its centre, see Fig. 1b. Note that in most cases, application of MFC with interdigitated electrode (IDE), where the polarisation direction is along the fibre length, is characterised by a low efficiency for energy harvesting comparing to a mono-fiber piezo-ceramic element (PZT) [23–25]. In this case the M8585-P2 device is polarised through thickness by continuous upper and lower electrodes. Compared to an IDE based device such a configuration has (1) a more uniform electric field distribution (2) a high device capacitance, leading to low peak voltages as a result of the piezoelectric charge and (3) a low electrical impedance due to the high device capacitance. The MFC has also an advantage in better flexibility. This property is crucial in our system as the axis of bending is changing due to bistability of a plate.

## Experimental results

**Fig. 2:**
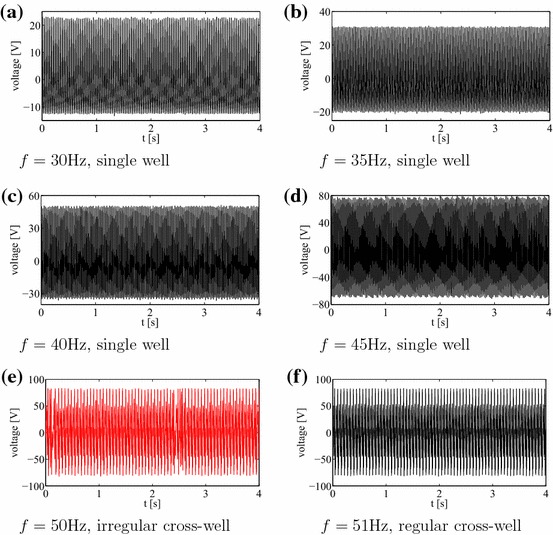

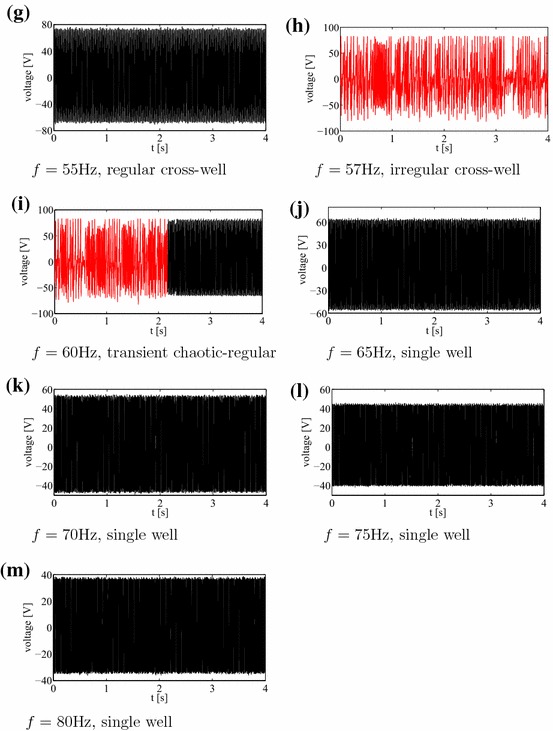
Voltage time series of experimental results corresponding for increasing frequencies $f=$ 30, 35, 40, 45, 50, 51, 55, 57, 60, 65, 70, 75, 80 Hz corresponding to subplots (**a**–**m**), respectively. Note single well mode cases (**a**–**d** and **j**-**m**), and snap-through buckling cases: regular (**f**, **g**), chaotic (**e**, **h**), and transient chaotic-regular (**i**). Stationary and transient chaotic responses are denoted by *red colour* for better clarity. Each excitation had 10*g* amplitude acceleration. Sampling frequency was 1000 Hz

Under kinematic excitation the laminate plate can show a variety of responses reflected in the measurement of the open-circuit voltage. In Fig. 2 we show the results for a sampling frequency of 1000 Hz.

The voltage-time response as a result of vibration testing with a 10*g* peak acceleration at frequencies ranging from of 30–80 Hz are summarised in Fig. 2. Interestingly, the voltage-time response exibits both periodic and non-periodic (chaotic) behaviour. In essence the results can be classified as

1. single well oscillations with no snap-through at small amplitude oscillations, as seen in Fig. 2a–d and j–m,
2. cross-well oscillations with snap-through at regular intervals (but not every cycle, Fig. 2g),
3. cross-well oscillations with snap-through at irregular intervals chaotic Fig. 2e, h,
4. continuous snap-through at every cycle, Fig. 2f.

**Fig. 3:**
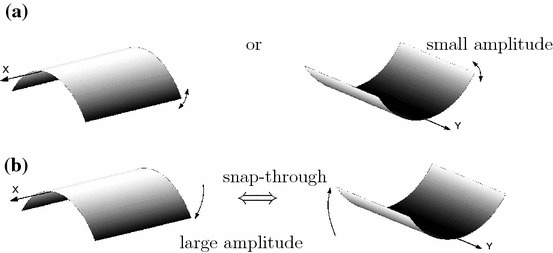
Vibration modes of a bistable square plate **a** single well: small amplitude vibrations around one of the equilibrium states; **b** snap-through: large amplitude vibrations

**Fig. 4:**
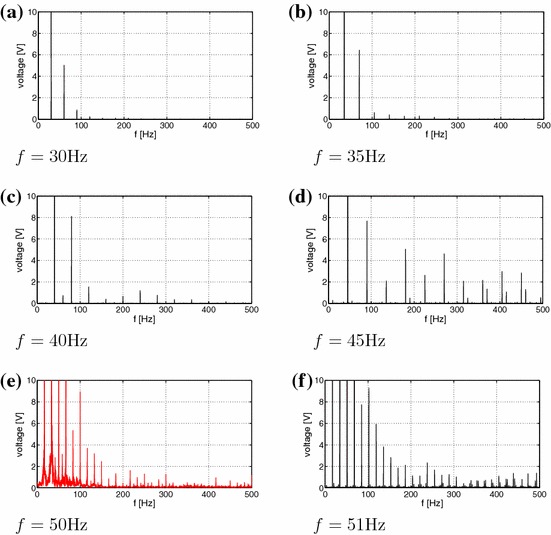

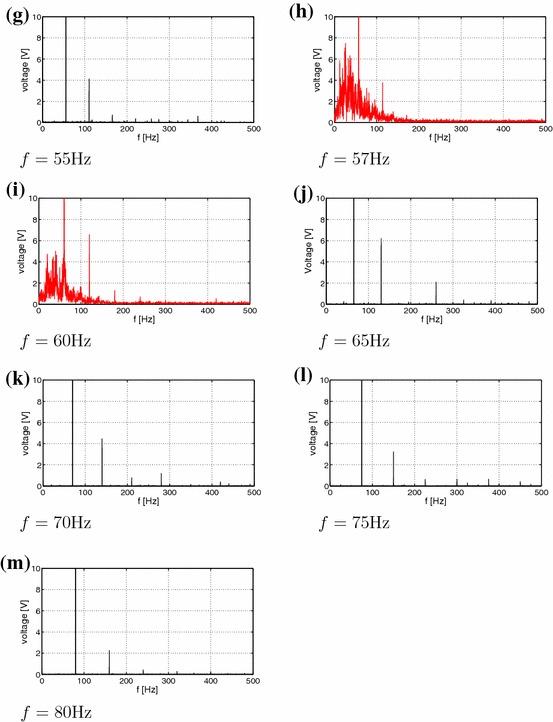
Frequency spectra (**a**–**m**) corresponding to voltage time series Fig. 2a–m, respectively

For better clarity the stationary chaos and transient chaotic responses are denoted by red colour. Figure 2i presents an interesting case where there is transient chaotic-regular behaviour. Schematic images of the possible mode shapes during single well and snap-through are shown in Fig. 3. Figure 4a–m shows the corresponding Fourier transforms of the examined measured voltage output. One can observe that the excitation frequency is accompanied by the higher harmonics in all the figures. In addition, in Fig. 4e, h, i there is smearing of the discrete frequency response into bands as expected for chaotic cases. Note that the frequency spectra represent a qualitative criterion of the system response. In the discussion below, the nature of the dynamic behavior of the responses will be explained using established tools.

**Fig. 5:**
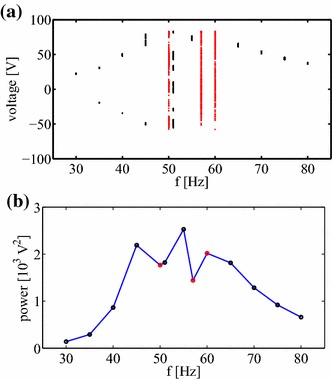
Bifurcation diagram (**a**)—created on the basis of local maximum points of the corresponding time series (Fig. 2), resonance curve (**b**)—${\text{var}}(u)$ versus frequency $f$. Note, the *red points* correspond to chaotic oscillations (see $f=50$, 57, and 60 Hz)

Interestingly, the more complex response cases are close to the resonance region. This has been summarised in Fig. 5a which is a bifurcation map created from the local maxima collected from cycles in the corresponding votage time series (Fig. 2). One can distinguish the regular and chaotic responses as singluar points and point bands, respectively. Note the case *f* = 51 Hz is not a clear case and has been clasified as a multifrequency regular case because of the discrete Fourier spectrum (see Fig. 4f). The associated resonance curve is estimated via the voltage output variance ${\text{var}}(u) = \sigma _u^2$ which is plotted versus frequency $f$ (Fig. 5b). Note that the large voltage response is acompanied by cross-well oscillations of regular and chaotic nature. Interestingly, chaotic oscillations are characterised by a smaller voltage output (see red points in Fig. 5b).

In the next sections, we propose to use the 0–1 test for more accurate chaos identification.

## The ‘0–1 test’

The ‘0–1 test’, invented by Gottwald and Melbourne [26, 27], can be applied for any system of a finite dimension to identify the chaotic dynamics but it is based on the statistical properties of a single coordinate only. Thus it is suitable to quantify the response where only one parameter was measured in time. As it is related to the universal properties of the dynamical system such as spectral measures, it can distinguish a chaotic system from a regular one.

A particular advantage of the 0–1 test over the frequency spectrum is that it provides information regarding the dynamics in a single parameter value, similar to the Lyapunov exponent. However, the Lyapunov exponent can be difficult to estimate in any non-smooth simulated or measured data [31]. The present system (Fig. 1) used an asymmetric bistable laminate plate as an example showing non-linear elastic properties. Therefore the 0–1 test can provide the suitable algorithm to identify the chaotic solution [32–35].

Starting from the voltage output $u(i)$, for sampling points $i=1,\ldots,N_t$, (where $N_t=4000$) we define new coordinates $p(n)$ and $q(n)$ as

1 $$\begin{aligned} p(n)= & {} \sum _{j=0}^{n} \frac{(u(j) - \overline{u})}{\sigma _u} \cos (j c),\\ q(n)= & {} \sum _{j=0}^{n} \frac{(u(j)- \overline{u})}{\sigma _u} \sin (j c), \end{aligned}$$

where $\overline{u}$ denotes the average value of $u$ while $\sigma _u$ its standard deviation, $c$ is a constant $\in [0,\pi ]$. Note that $q(n)$ is a complementary coordinate in the two dimensional space. Furthermore, starting from bounded coordinate $u(i)$ we build a new series of $p(n)$ which can be either bounded or unbounded depending on dynamics of the examined process.

Continuing the calculation procedure, the total mean square displacement is defined as

2 $$\begin{aligned} M_c(n)& =  {} \lim _{N \rightarrow \infty } \frac{1}{N} \sum _{j=1}^N [ \left( p(j+ n) - p(j)\right) ^2 \\&\quad+\, \left( q(j+ n) -q(j)\right) ^2 ], \end{aligned}$$

The asymptotic growth of $M_c(n)$ can be easily characterized by the corresponding ratio $K'_c(n)$

3 $$\begin{aligned} K'_c(n)= \frac{\ln (M(n))}{\ln n}. \end{aligned}$$

In the limit of a very long time $$n \rightarrow \infty $$ (in practice $$n =n_{max}=400$$ while $$N=3600$$) we obtaine the corresponding values of $$K_c$$ for a chosen $$c$$ value. Note, our choice of $$n_{max}$$ and $$ N$$ limits (in Eqs. 4 and 5) is consistent with that proposed by Gottwald and Melbourne [28–30] $$ N, n_{max} \rightarrow \infty $$ but simultaneously $$n_{max}$$ should be about $$N/10$$.

It is important to note that the parameter $c$ acts like a frequency in a spectral calculation. If $c$ is badly chosen, it could resonate with the excitation frequency or its ultra- or sub-harmonics. In the 0–1 test regular motion would yield a ballistic behaviour in the $(p,q)$-plane [28] and the corresponding $M_c(n)$ results in an asymptotic growth rate even for a regular system. The disadvantage of the test, its strong dependence on the chosen parameter $c$, can be overcome by a proposed modification. Gottwald and Melbourne [28, 33, 34] suggest to take randomly chosen values of $c$ and compute the median of the corresponding $K_c$-values.

Consequently, the new covariance formulation

4 $$\begin{aligned} K_c= \frac{\text{cov}({\mathbf{X}},{\mathbf{M}}_c)}{\sqrt{\text{var}({\mathbf{X}}) \text{var} ({\mathbf{M}}_c})}, \end{aligned}$$

where vectors ${\mathbf{X}}\,=\,[1, 2, \ldots, n_{max}],$ and ${\mathbf{M}}_c\,=\,$[$M_c(1)$, $M_c(2)$, …, $M_c(n_{max})$].

In the above, the covariance $\text{cov}({\mathbf{x}}, {\mathbf{y}})$ and variance $\text{var}({\mathbf{x}})$, for arbitrary vectors ${\mathbf{x}}$ and ${\mathbf{y}}$ of $n_{max}$ elements, and the corresponding averages $\overline{x}$ and $\overline{y}$ respectively, are defined

5 $$\begin{aligned} \text{cov}({\mathbf{x}},{\mathbf{y}})= & \,\frac{1}{n_{max}} \sum _{n=1}^{n_{max}} (x(n)-\overline{x})(y(n)-\overline{y}),\\ \text{var}({\mathbf{x}})= & \,\text{cov}({\mathbf{x}}, {\mathbf{x}}). \end{aligned}$$

Finally, the median is taken of $K_{c}$-values (Eq. 6) corresponding to 100 random values of $c \in (0,\pi )$. Such an average $K$-value can now be estimated for various excitation frequency $f$. The control parameter $K$ signals the appearance of regular and chaotic solution for $K$ close to 0 and one, respectively.

## Regular and chaotic oscillations by the ‘0–1 test’

**Fig. 6:**
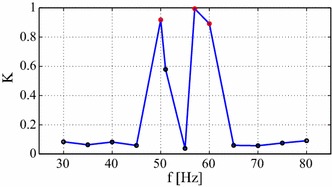
Control parameter of the 0–1 test, $K$ (c). $K \rightarrow 0$ indicates a regular solution while $K \rightarrow 1$ signals chaos. Note, the *red points* correspond to chaotic oscillations (see $f=50$, 57, and 60 Hz)

The results of the parameter $K$ are presented in Fig. 6. We show that for chaotic regions $K \ge 0.9$ while for regular regions $K$ is closer to 0 ($K \le 0.1$). The case of intermediate value (see $K=0.58$ for $f=$51 Hz in Fig. 6) signals the vicinity to bifurcation points (see $f=$51 Hz in the bifurcation diagram Fig. 5a) or a very long transient. In such cases a longer time series can outweigh the classification assignment to a regular response.

**Fig. 7:**
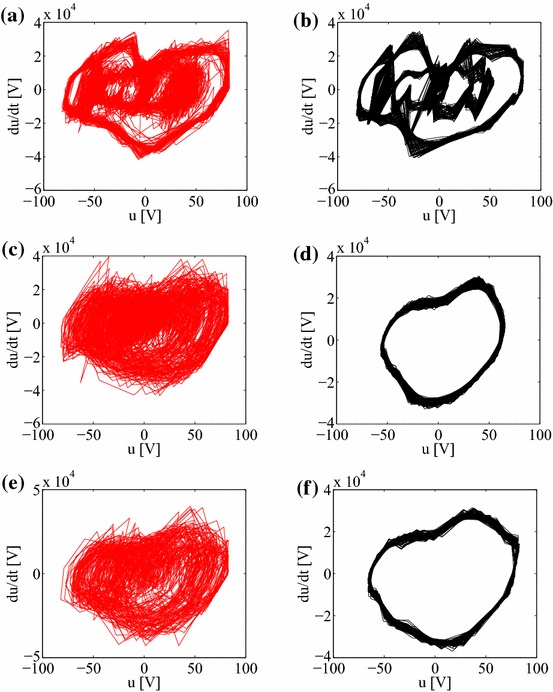
Phase portraits $du/dt$ versus $u$ obtained by numerical differentiation for chosen voltage time series at frequencies **a** $f=$50 Hz ($K= 0.918$), **b** 51 Hz ($K=0.579$), **c** 57 Hz ($K=0.994$), **d** 65 Hz ($K=0.059$); and finally the transient chaos versus regular cases (see Figs. 2, 6) $f=60$ Hz ($K= 0.892$) plotted in (**e**) and (**f**), respectively

**Fig. 8:**
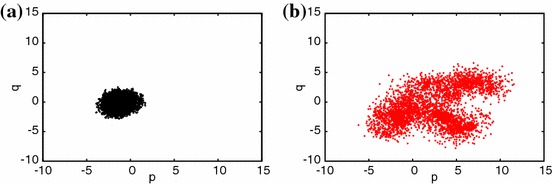
Phase plane in $(p,q)$-coordinates for $f=55$ Hz (regular—**a**) and $f=57$ Hz (chaotic—**b**) estimated for $c=1$. The corresponding values of $K_c$ were estimated as $K_c=0.066$ and $K_c=0.988$ for cases **a** and **b**, respectively

For selected chaotic and neighbour frequency cases we now plot the corresponding phase portraits (Fig. 7). It is possible to differentiate regular responses as the close orbits patterns in contrast to strange chaotic attractors. Interestingly, the transient case shows the clear difference between the initial (transient chaotic) and final (regular) behaviour (Fig. 7e, f, respectively). For better clarity we also show examples of the phase plane in the new $(p,q)$ coordinates (Eq. 1). Figure 8a, b shows the growth of the displacement in regular (Figs. 2f, 4f) and chaotic (Figs. 2f, 4f) solutions suggesting the bounded and unbounded cases. The corresponding values of $K_c$, $K_c=0.066$ and $K_c=0.988$, distinguish unambiguously the regular and chaotic cases.

## Conclusions

The dynamics of a CFRP bistable laminate combined with a piezoelectric MFC has been examined and the existence of chaotic responses have been successfully identified using the 0–1 test. The results obtained are consistent with quantitative methods such as Fourier frequency spectra and corresponding phase portraits. Note that the present investigations are contaminated by a relatively small measurement noise level which is present in any experimental data. This is visible in the values of $K \approx 0.1$ for regular responses. However, better convergence with $K \rightarrow 0$ or 1 was achieved indicating that a distinction between regular and chaotic motion could be achieved if a longer time series was applied. It is also noted that due to the elastic non-linear properties of the examined system a relevant quantitative characterisation (via Lyapunov exponents) of responses is difficult. A further study may involve more sophisticated time-series approaches with a suitable dimensional space embedding [36].

Note that the above identification could be useful for optimising the energy harvester response to a specific vibration input. By focusing on the resonance region (by comparing Figs. 5a, b, 6) it is possible to observe a less complex motion (smaller $K$) leading to the higher variance of the voltage output [higher var(u)]. For example local minima in Fig. 5b are correlated with peaks in Fig. 6 for the same frequencies $f =45$ and 55 Hz. Further studies and needed to draw a more general conclusion on the relationship between the power output and $K$.
